# Insight into Polyphenol and Gut Microbiota Crosstalk: Are Their Metabolites the Key to Understand Protective Effects against Metabolic Disorders?

**DOI:** 10.3390/antiox9100982

**Published:** 2020-10-13

**Authors:** Mireille Koudoufio, Yves Desjardins, Francis Feldman, Schohraya Spahis, Edgard Delvin, Emile Levy

**Affiliations:** 1 Research Centre, Sainte-Justine University Health Center, Montreal, QC H3T 1C5, Canada; mireille.koudoufio@umontreal.ca (M.K.); francis.feldman@umontreal.ca (F.F.); schohraya.spahis@gmail.com (S.S.); delvine@sympatico.ca (E.D.); 2 Department of Nutrition, Université de Montréal, Montreal, QC H3T 1J4, Canada; 3 Institute of Nutrition and Functional Foods, Laval University, Quebec City, QC G1V 0A6, Canada; yves.desjardins@fsaa.ulaval.ca; 4 Department of Biochemistry, Université de Montréal, Montreal, QC H3T 1J4, Canada; 5 Department of Pediatrics, Université de Montréal, Montreal, QC H3T 1J4, Canada

**Keywords:** dietary polyphenols, metabolites, oxidative stress, inflammation, epigenetics, microflora, cardiometabolic complications

## Abstract

Lifestyle factors, especially diet and nutrition, are currently regarded as essential avenues to decrease modern-day cardiometabolic disorders (CMD), including obesity, metabolic syndrome, type 2 diabetes, and atherosclerosis. Many groups around the world attribute these trends, at least partially, to bioactive plant polyphenols given their anti-oxidant and anti-inflammatory actions. In fact, polyphenols can prevent or reverse the progression of disease processes through many distinct mechanisms. In particular, the crosstalk between polyphenols and gut microbiota, recently unveiled thanks to DNA-based tools and next generation sequencing, unravelled the central regulatory role of dietary polyphenols and their intestinal micro-ecology metabolites on the host energy metabolism and related illnesses. The objectives of this review are to: (1) provide an understanding of classification, structure, and bioavailability of dietary polyphenols; (2) underline their metabolism by gut microbiota; (3) highlight their prebiotic effects on microflora; (4) discuss the multifaceted roles of their metabolites in CMD while shedding light on the mechanisms of action; and (5) underscore their ability to initiate host epigenetic regulation. In sum, the review clearly documents whether dietary polyphenols and micro-ecology favorably interact to promote multiple physiological functions on human organism.

## 1. Introduction

Polyphenols, synthesized in a wide variety of fruits, legumes, and herbs, interestingly serve to protect against biotic and abiotic stresses caused by insects and parasites [1,2,3]. However, much attention is presently given to dietary polyphenols in view of their evident health benefits. These phenolic compounds are intrinsically strong free radical scavengers [4] and exhibit obvious potential in alleviating oxidative stress (OxS)-related illnesses. In this context, epidemiological and animal food intervention studies have established a solid association between the consumption of polyphenol-rich foods or beverages and their preventive influence on complex diseases, including insulin resistance (IR) [5,6,7,8], type 2 diabetes (T2D) [9,10,11,12], obesity [5,9,13,14,15,16], cardiovascular diseases (CVD) [17,18,19,20,21,22], neurodegenerative disorders [23,24,25,26], and cancer [22,27,28,29]. Recently, polyphenols have also been suggested as plausible adjunctive therapeutic agents for the COVID-19-induced inflammatory storm [30,31,32].

The growing evidence as to the prevention and management of different disorders by dietary polyphenols has led to multiple studies focusing on their dietary sources [33], efficacy [20], and bioavailability [34,35,36]. However, much remains to be learned about their potential mechanisms of action. In particular, their dynamic interaction with intestinal microbiota is of utmost importance as ~90% of the amount ingested reach the colon to directly influence microbial ecology and, at the same time, undergo microflora-induced metabolic modifications [37,38]. These interactions represent the specific topic of the present critical review. First, we will discuss the prebiotic action of polyphenols. Second, we will focus on their transformation and physiological impact on the gastrointestinal (GI) tract. Third, we will emphasize the role of their metabolites resulting from their catabolism by colonic bacteria. Fourth, we will analyze their cardiometabolic effects while highlighting their mechanisms of action and underscoring epigenetic regulation. Clearly, the ultimate goal of this review is to show that the health benefits of dietary polyphenols are through the action of their bioactive metabolites. Nonetheless, for the reader’s understanding, it is essential to provide a brief introduction, which describes the structure, classification, and bioavailability of polyphenols.

## 2. Polyphenols: Classification, Structure, and Bioavailability

### 2.1. Classification and Structure of Polyphenols

Concisely, polyphenols are characterized by at least one aromatic ring and one hydroxyl functional group with evolving structure from a simple molecule to a complex polymer. They are classified into flavonoids and non-flavonoids, according to the intricacy of their structure, number of phenol rings, and carbon skeleton [32,39,40,41].

Flavonoids present a benzo-γ-pyrone structure containing two aromatic rings (A and B) bound by a 3-carbon bridge (C6–C3–C6). Based on the differences in the C ring, flavonoids can be subdivided into six sub-classes, namely: (1) flavonols (e.g., quercetin, kaempferol); (2) flavones (e.g., luteolin, apigenin); (3) isoflavones (e.g., daidzein, genistein); (4) flavanones (e.g., naringenin, hesperetin); (5) flavanols (e.g., catechins, epigallocatechins); and (6) anthocyanidins (e.g., malvidin, cyanidin). Although polyphenols may be encountered in plants as aglycones, they are generally found as glycoside derivatives, glucoside, galactoside, rhamnoside, xyloside, rutinoside, arabinopyranoside, and finally arabinofuranoside, being the most common [39]. Interestingly, the various tannin functional groups (e.g., hydroxyls) allow them to create non-covalent (hydrophobic and hydrogen) bounds and covalent bonds with proteins and carbohydrates [42,43,44]. Moreover, tannins are polyphenols that are most involved in binding to proline-rich proteins [45].

### 2.2. Bioavailability of Polyphenols

#### 2.2.1. Factors Affecting Polyphenol Absorption

Polyphenol bioavailability (e.g., alimentary proportion delivered to blood circulation) is dependent on diverse conditions such as their stability, transport, and metabolic behavior. Several intrinsic and extrinsic factors influence the content of plant polyphenols [35]. It is worth mentioning that their relative composition and levels vary extensively among species and between varieties of the same species in association with their genetic background and state of ripeness, which is related to the time of harvest. For example, the content of olive secoiridoid phenolic derivatives decreased when irrigation and ripening increased [35,46,47,48]. Furthermore, the concentration and variety of phenolic derivatives depend on storage conditions as illustrated by the variability of the phenolic content and total antioxidant capacity of 14 apple and 6 pear cultivars harvested at different periods [46]. The chemical structure is another important intrinsic factor when considering bioavailability. Polyphenols vary widely in molecular weight, secondary and tertiary structures, as well as glycosylation level [49,50,51]. For instance, resveratrol and quercetin glycosides are absorbed to a lesser extent than the respective aglycones, which influences their metabolic fate through the digestive tract, their absorption and release in the portal circulation, and excretion in urine or feces [52].

With regards to extrinsic aspects, short time storage at low temperature also affects polyphenol content as exemplified by the broccoli loss of its caffeoyl-quinic and sinapic acid contents [53]. Food processing is yet another external factor when considering polyphenol bioavailability. Thermal processing causes diverging effects on polyphenol content and absorption. For instance, Xu et al. [54] reported that thermal processing significantly decreased bean total phenolic content and antioxidant properties, while Khatun et al. [55] reported the opposite by documenting an increase in the same properties when heating spices. These dissimilarities may be explained both by the difference in polyphenol moieties, matrix, and cooking process. The role of the food matrix on the disposition of polyphenols should not be under-evaluated given the interaction between food components ingested simultaneously (fat, proteins, complex carbohydrates) and different polyphenolic compounds likely influencing their interaction with the microbiota and ultimately their absorption. In a rat model, the combination of lecithin and soya bean oil or emulsifiers (sucrose, fatty ester, polyglycerol fatty acid ester, and sodium taurocholate) increased intestinal absorption efficiency of water-dissolved quercetin when these constituents had no effect given separately [56]. Similarly, the administration of hydroxytyrosol as a sole natural product to humans or rats provides a better bioavailability than when combined with refined oil or yogurt, although its handling differed between the two species [57]. Differing digestive tract possesses such as luminal machinery, bile acid secretion, and enterohepatic cycle may explain the differences. The above examples suffice to warrant precautions when interpreting results obtained from different protocols. The several factors mentioned, and others have to be carefully taken into account when conducting studies using cellular models, animals, or clinical investigations, as they could explain variability in outcomes results from numerous reports. In general, polyphenols exhibit a low bioavailability with maximal plasma concentrations reached within 2 to 4 h post ingestion, and an apparent short elimination time with a return to baseline levels within 8 to 12 h [58]. Hence, 24 h-urine collections generally provide a more accurate evaluation of total polyphenol absorption, metabolism, and excretion [59]. These pharmacokinetic properties suggest that only long-term consumption of a variety of polyphenols will affect the health trajectory and support the role of the “Mediterranean diet” in improving population health perspectives.

#### 2.2.2. Absorption of Polyphenols and Derivatives

**Figure 1:**
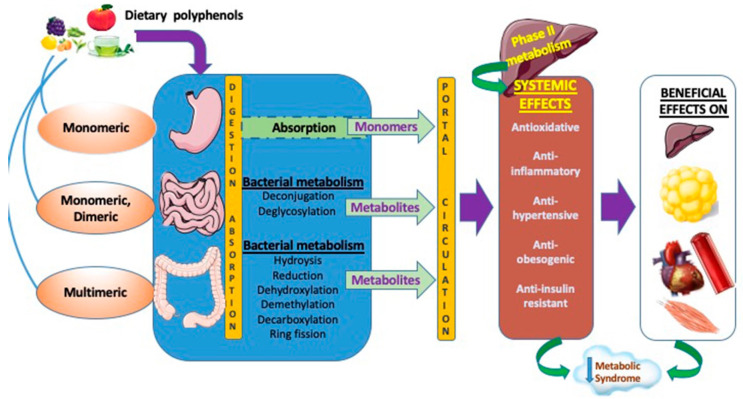
Integrated view of intestinal dietary polyphenol absorption, luminal transformation, and actions of their relevant metabolites on cardiometabolic disorders. As dietary polyphenols have limited absorption in the stomach and the small intestine, the unabsorbed polyphenols continue their transit to the colon where they are hydrolyzed, demethylated, decarboxylated, dehydroxylated, and ring fissioned by microbiota. Following these processes, microbial metabolites are subjected to phase II metabolism in the colon and liver, and enter the bloodstream to exert their biological effects, which extend to peripheral organs. Unabsorbed polyphenols and metabolites are excreted in feces, and absorbed microbial metabolites are mostly excreted in the urine. Noteworthy, whereas polyphenols improve microbiota composition, diversity and functions through their prebiotic actions, gut microbiota transform them into efficient bioactive regulators capable of optimizing cardiometabolic health in healthy individuals, while alleviating and mitigating the metabolic syndrome in patients. *Created with Servier Medical Art (A service to medicine provided by Les Laboratoires Servier, 50, rue Carnot—92284 Suresnes Cedex—France.

Polyphenol intestinal absorption was indirectly estimated by raised antioxidant defense in the circulation in response to their consumption. The basic principles for their transport and local metabolism are partway established. Figure 1 schematizes the digestion and absorption processes as generally accepted today for polyphenols. Distinction must be made between the digestive and absorption processes of low and high molecular weight polyphenols along the digestive tract as the sites and the efficacy may differ. In decreasing order of absorption kinetics, isoflavones, caffeic and gallic acids lead, followed by catechins, flavanones, and quercetin glucosides. High molecular weight polyphenols such as proanthocyanidins, galloylated catechins, and anthocyanins come last [60]. For instance, the degree of polymerization of procyanidins (dimer B3, trimer C2, and polymers), isolated from willow tree catkins, decreased their absorption through the rat gut barrier and can limit their metabolism by the intestinal microbiota when compared to catechins [61].

#### 2.2.3. Gastric Uptake

It is well established that plant polyphenols predominantly present as glycosides undergo deglycosylation to their respective lipophilic aglycones, thereby enhancing their absorption through the GI [62,63]. Whether the stomach plays a significant role remains uncertain until today. To our knowledge, there is little direct in vivo evidence on the contribution of the human stomach to the assimilation of phenolic compounds. It needs to be underlined that in vitro simulation or animal models are the most common sources of information. Table 1 summarizes the results obtained in in vitro and in vivo models as well as in humans. 

Purified cocoa procyanidin oligomers, incubated in simulated gastric juice, led to the appearance of dimers and monomers in a time-dependent fashion [64,65]. Similarly, olive oil polyphenol glycosides, including oleuropein (glycoside of elenolic acid linked to hydroxytyrosol) underwent rapid and time-dependent hydrolysis, yielding appreciable amounts of free hydroxytyrosol and tyrosol, probably through nucleophilic attack [66]. On the contrary, other studies, using the same in vitro model, along with oral administration of polyphenols to humans, showed stability of a variety of polyphenols in the acidic milieu [67,68,69,70,71,72,73]. Interestingly, the incubation of apple phloretin and quercetin for 5 min with native saliva resulted in the production of the respective aglycones, likely by oral bacterial flora as the process was blocked by antibiotics [68]. In the same study, hydroxycinnamic acid derivatives, flavonols, dihydrochalcones, and monomeric flavan-3-ols were stable when incubated with simulated gastric juice at pH 1.8, but procyanidin B_2_ was degraded. Obviously, the in vitro models suffer from being an inadequate reflection of the gastric fluid, which lacks mucus normally secreted by parietal cells. This is supported by the notable stability of cocoa procyanidin polymers in vivo in the human stomach environment [73]. In terms of in vivo studies, the in situ digestion rat pylorus ligated model shows that polyphenol aglycones are absorbed by the stomach, and suggests the limited role of the stomach in flavonoid glycosides metabolism [74,75,76,77]. These results reveal inequality in the behavior of polyphenols in different experimental models and warrant careful analysis to obtain a complete representation.

#### 2.2.4. Small Intestine Uptake

##### Duodenum

Using the in vitro three-step model simulating the digestive process from the mouth to the small intestine [78], it was shown that the duodenal digestion phase either with static or continuous-flow cellulose membrane-based dialysis containing bile salts and pancreatin resulted in dialyzable (chyme-available for passive absorption into the systemic circulation) and non-dialyzable (digested fraction available for colon) fractions [79]. Profiling the chyme and the non-dialyzable procyanidin content of cocoa liquor post gastric digestion revealed that the high-molecular weight procyanidins (pentamers to nonamers) were hydrolyzed essentially into monomers and dimers. Similar profiles were observed for the duodenal digest. Interestingly, the cocoa liquor duodenal digest contained much higher procyanidin concentrations than the cocoa powder counterpart that the authors attributed to the protective effect of fat micellar structures present in the liquor. More recently, using the same dynamic duodenal model, most of the intact procyanidins isolated from chocolate nibs were retained in the non-dialyzable fraction, which would likely be available for colonic digestion by the microbiota, while smaller molecular weight dimers and trimers were quantified in the dialyzable fraction [67]. On the other hand, the addition of carbohydrate-enriched food resulted in lower duodenal digestibility of procyanidin. Last, with the ex vivo reverted duodenal sac model, the bioavailability of curcumin was found to be significantly enhanced when encapsulated in low- and high-molecular weight polylactic-co-glycolic acid nanoparticles, possibly attributable to faster dissolution following the administration [80]. Overall, these results underpin the effect of the food matrix on the metabolism of polyphenols.

##### Jejunum and Ileum

It is well known that under physiological conditions, the small intestine, particularly the jejunum and ileum segments, represents the principal location for the digestion and absorption of dietary lipids. This holds for polyphenols, although with differing efficiency according to their molecular weight and type of glycosylation. Upon reaching the jejunum and ileum, glycosylated polyphenols are either hydrolyzed into their respective aglycones by the membrane-bound brush-border lactase phlorizin hydrolase [81] or transported intact by the enterocyte sodium-glucose co-transporter and hydrolyzed by the cytosolic β-glucosidase [82,83,84]. The nature of carbohydrate moieties affects the absorption of polyphenols through the small intestine. For instance, whereas glucoside conjugates and their aglycones are absorbed in the small intestine, those containing rhamnose molecules, [e.g., the flavonols hesperidin (hesperidin-7-*O*-glucosyl-rhamnose) and rutin (quercetin-3-*O*-glucosyl-rhamnose)], must proceed to the colon where rhamnose will be removed by bacterial rhamnosidase [85]. On the other hand, catechin and epicatechin, monomers of the flavonol family, which are often acylated by gallic acid, are readily absorbed by enterocytes without any deconjugation or hydrolysis [86,87].

Caco-2 cells, a human colon carcinoma derived cell line, which upon confluence develops enterocyte-like characteristics, have extensively been used as a model for studying lipid and drug metabolism [88] and transport [89]. They also have served as a model for studying the absorption, transport, and metabolism of polyphenols as detailed in Table 2.

Caco-2 cells have been shown to absorb major dietary hydroxycinnamates and diferulates after de-esterification (phase 1 transformation) and conversion into glucuronate, methyl, and sulfate conjugates (Phase 2 transformation) [90]. In 2006, Corona et al. [66] reported that Caco-2 cells transported hyroxytyrosol from the apical to the basolateral compartment, with the appearance of 3-*O*-methyl–hydroxytyrosol and glutathionyl-hydroxytyrosol in both compartments. They observed a similar transport for tyrosol, without any evidence of further metabolism or transport for oleuropein. The same year, trans-epithelial transport of trans-piceid (3-β trans-resveratrol glucoside) and deglycosylation in trans-resveratrol were reported using human intestinal Caco-2 cell monolayers [82]. The important question in the context of nutrition is whether polyphenols behave similarly when evaluated as single compounds as when assessed in complex mixtures [93,94]. Su et al. [92] partially answered this question by showing that, in Caco-2 cells, (+)-catechin significantly enhanced the cellular uptake and transport of the isoflavone daidzein-8-*C*-glucoside, whereas this compound significantly abated that of (+)-catechin, and that inhibition of efflux pumps increased their uptake. Although valuable for screening polyphenol bioavailability, caution should be used when interpreting the data obtained from the Caco-2 cellular model in terms of predictability. Indeed, even if in vitro smaller phenolic derivatives of hesperidin 2S were absorbed, hesperidin conjugates were the main bioavailable moieties in a randomized human controlled study [69].

The intestinal porcine enterocyte cell line (IPEC), a non-transformed non-tumorigenic permanent intestinal cell line derived from the jejunum is another model that merits attention for two main reasons. First, it maintains its differentiated characteristics, and second, there is a close analogy in results obtained in vivo with the porcine model, the closest to the human GI system [95,96]. To our knowledge, it has seldom been used to study polyphenol absorption. However, a good correlation for the uptake of grape pomace polyphenols was observed between IPEC cells and the duodenum and colon of piglets [97]. In addition, Wan et al. [98] proved functionality of the same in vitro model by showing enhanced the secretion of α-defensins-1 and 2 and reduced the E. coli translocation across a confluent IPEC cells by epigallocatechin-3-gallate, the major polyphenol specie in green tea.

##### Colon

The case of the colon bears some singularity in the sense that there is a reciprocal relationship between polyphenols and colonic microbiota. It has been clearly established that the gut microbiome is a key actor in modulating the production, bioavailability, and biological activities of complex low molecular weight phenolic metabolites grouped under the term polyphenol metabolome [99,100]. Rarely, the transport of polyphenols by the colon has directly been evaluated as most of the studies employed intestinal cell models displaying features of the small intestine [101]. It is, however, worthy to mention that T84 colon carcinoma cell line monolayers, incubated with ferulic, isoferulic, cinnamic, and hydroxycinnamic acids, as well as with flavonoids, showed appreciable transport from the luminal to the basolateral side [102]. Measurable levels of ferulic glucuronide and sulfate were detected in the apical and basolateral compartments when supplied at supra-physiological concentrations. Other reports bear mostly on the polyphenol anticancerogenic properties without addressing the uptake and transport per se [103,104,105,106].

The enterohepatic cycle is an important mechanism for the bioavailability and disposition of polyphenol aglycones and their metabolites. This is true whether absorbed by the small intestine or the colon (where they undergo in situ phase II enzymatic conversion to their respective methyl, glucuronide, and sulfate conjugates) or delivered to the liver via the portal circulation for further conjugation (before entering the blood stream and being excreted in urine). Alternatively, they enter the bile duct where mixed with bile salts, re-enter the small intestine for either a second-round absorption by enterocytes and transport to the liver, or excretion in the stools [37,107,108,109].

## 3. Interaction between Polyphenols and the Colon Microbiota

Humans are colonized by a wide array of microorganisms referred to as the microbiota that consists of obligate and facultative anaerobes, mainly targeting the colon [110,111]. Their main assignment is to provide assistance to their host in digesting food complex polysaccharides and proteins unprocessed in the upper GI tract [112]. The saccharolytic pathway, mainly active in the proximal colon, yields short chain fatty acids, of which acetic, propionic, and butyric acids are the most abundant, as well as lactic acid, CO_2_, methane, and ethanol. The second catabolic pathway responsible for protein fermentation, mainly present in the distal colon, leads to metabolite species such as ammonia, various amines, thiols, phenols, and indoles [113,114]. The healthy gut ecosystem consists of the anaerobes *Bacteroidetes* and *Firmicutes*, the latter contributing to more than 90% of the total bacterial species, and *Proteobacteria*, *Verrucomicrobia*, and *Actinobacteria* accounting for the balance [115].

Diet composition has a definite role in the taxonomic and functional profile of the colon microbiota. For example, several reports have shown that high carbohydrate and fiber intakes are related to higher abundance of 3 enterotypes: *Lachnospiraceae*, *Ruminococcaceae*, and *Bifidobacteria* [116,117,118]. For their part, omnivorous women were shown to have a higher abundance of fecal butyrate producing taxa (*Clostridium cluster XIVa* and *Roseburia/Eubacterium*) than vegetarians [119]. Intervention studies have also established that changing from a carnivorous diet to a plant-based diet resulted in a gradual decrease in abundance of *Bacteroides*, a bile-tolerant symbiotic microorganism, and an increase in *Firmicutes* that preferentially metabolizes plant polysaccharides [120]. Furthermore, population food tradition-related differences in microbiota constitution have been reported; those consuming meat-based diets had higher abundances of *Bacteroides* than those traditionally consuming plant-based diets [118,121]. A controlled 10-week-diet intervention, involving overweight volunteers, demonstrated a rapid modification in microbiota species composition despite the inter-individual variation in initial composition [122]. Overall, the results indicate that *Bacteroides*, together with *Alistipes* and *Parabacteroides*, may be the primary proteolytic taxon in the human colon [112].

### 3.1. Polyphenol–Microbiota Interaction

**Figure 2:**
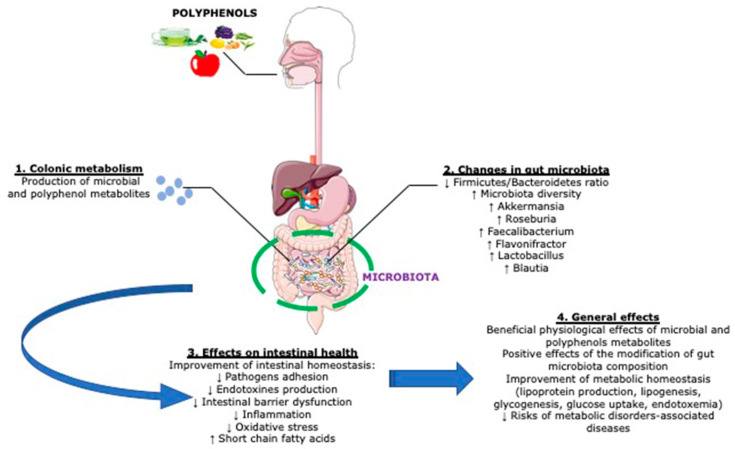
Beneficial actions resulting from the interaction between polyphenols and intestinal microbiota. Polyphenolic compounds exhibit prebiotic ability, which modifies bacterial composition and function. On the other hand, intestinal microbiota ameliorates intestinal homeostasis and cardiometabolic health by producing polyphenolic metabolites. * Created with Servier Medical Art.

Of interest, although still poorly understood, evidence is accruing on the influence of dietary polyphenols, perceived as xenobiotics, on the modulation of the colonic microflora and health [123]. They interfere with bacterial cell-to-cell communication and coordinate pathogenic behaviors, two clinically important characteristics with regards to virulence control and wound healing [124,125]. They also sensitize bacteria to xenobiotics and alter membrane permeability. This is exemplified by the sensitization of methicillin-resistant *Staphylococcus aureus* to β-lactam through the (−)-epicatechin gallate-mediated alterations of the bacterium cell wall bilayer, and enhanced release of lipotechoic acid from the cytoplasmic membrane [126]. Polyphenols, acting as prebiotics, have also been shown to modulate gut metabolism and immunity as well as inflammatory pathways through the change of T-cell functions, inhibition of mast cell degranulation, and down regulation of inflammatory cytokine responses [127,128,129,130]. Studies in animals and in humans have shown modification of colon microflora by polyphenolic compounds, resulting in growth inhibition of certain bacterial groups while permitting others to flourish [131,132,133]. Valuable effects of polyphenols, such as those present in red wine, include among others, enhanced abundance of beneficial bacterium taxa such as *Bifidobacterium* and *Lactobacillus*, capable of improving gut barrier protection, *Faecalibacterium prausnitzii* having anti-inflammatory properties, and *Roseburia* as a butyrate producer. Interestingly, the proliferation of these bacteria occurred at the expense of the less desirable *Escherichia coli* and *Enterobacter cloacae* [134]. Most of the important biological actions resulting from phenolic compounds and microbiota are depicted in Figure 2.

### 3.2. Impact of Microbiota on Polyphenol Metabolism

The transformation of polyphenols into bioactive metabolites is tributary to the colonic microflora species. For example, the *Firmicutes*: *Eubacterium ramulus* and *oxidoreducens*, *Clostridium orbiscidens* and *Flavonifractor plautii* metabolize the flavonols Kaempferol, Quercetin, and Myricetin by *O*-deglycosylation and C-ring fission into a series of metabolites comprising protocatechuic, 2-(3,4-dihydroxyphenyl-acetic, 2-(4-hydroxyphenil)-propionic, 3-hydroxyphenylacetic, 3-(3-Hydroxyphenyl)-propionic, 2-(3-hydroxyphenil)-acetic, 2-(3-dihydroxyphenil)-acetic, and 3-(3,4-dihydroxyphenil)-acetic acids as well as short-chain fatty acids (SCFAs) [83,135,136] (Figure 2). Furthermore, *Enterococcus casseliflavus (Firmicutes)* hydrolyzes sugar moieties from quercetin-3-*O*-glucoside, a process releasing the aglycone and ultimately producing lactic, formic, and acetic acids, together with ethanol [137]. The *Actinobacteria*: *Slackia equolifaciens*, *Slackia isoflavoniconvertens*, *Eggerthella lenta*, *Adlercreutzia equolifaciens*, and *Bifidobacterium* spp metabolize the flavonones Naringenin and Hesperidin through C-ring fission yielding 3-(4-hydroxyphenyl)-propionic, 3-phenylpropionic and hydroxyphenylpropionic acids [135,136]. *Adlercreutzia equolifaciens*, *Paraeggerthella hongkongensis*, *Slackia equolifaciens*, *Slackia isoflavoniconvertens*, *Bacteroides ovatus*, *S. intermedius*, *R. productus*, *E. sp. Julong*, *E. faecium EPI1*, *L. mucosae EPI2*, and *F. magna,* have also been shown convert some the isoflavone daidzein to (*S*)-equol, a nonsteroidal estrogen-like molecule [((3*S*)-3-(4-hydroxyphenyl)-7-chromanol)] [108,137].

## 4. Polyphenols and Metabolic Syndrome

The polyphenol metabolites, produced by the gut microbiota once absorbed and directed to the target tissues and organs, contribute to the metabolic health, through their antioxidant and anti-inflammatory properties, by preventing or reducing the risk of developing several cardiometabolic disorders (CMD), notably the metabolic syndrome (MetS).

The increasing prevalence of childhood MetS along with overweight and obesity is a major public health concern in both developed and developing countries. MetS is a key risk factor for the development of T2D, non-alcoholic fatty liver disease, and CVD in early adulthood [138,139,140]. MetS is a cluster of interrelated risk factors, including abdominal obesity, dyslipidemia, hypertension, hyperglycemia, and IR, which is triggered by cellular redox imbalance and inflammation as cardinal features [141,142,143].

In the last two decades, strategies to limit the deleterious effects of the MetS have been focused on diet regimen containing natural fruits, green vegetables, whole grains, legumes, probiotics, vitamin C, vitamin E, and ω-3 polyunsaturated-fatty acids [20,144,145,146,147,148]. Likewise, prebiotics, including polyphenols with their prebiotic potential, have also demonstrated promising effects on IR, glycemia, lipid profile, and CVD [8,134,149]. Other multiple studies, involving animal models, have consistently shown that administration of polyphenols, extracted from various sources, increased insulin sensitivity (via measurement of homeostatic model assessment for insulin resistance index, glucose intolerance, or insulin tolerance tests) and decreasing circulating free-fatty acid, triglyceride, cholesterol, C-reactive protein, resistin, and leptin concentrations [150,151,152,153,154]. At the clinical level, a parallel, double-blinded controlled and randomized 6-week dietary intervention demonstrated that strawberry and cranberry polyphenols improved insulin sensitivity in overweight and obese nondiabetic, insulin-resistant human subjects [8]. There was not a significant improvement of other cardiometabolic risk factors, which is probably due to the length of the intervention. Nevertheless, these in vivo results show the potential clinical preventive and therapeutic impact of polyphenols on human diseases. Considering that impaired redox potential and inflammatory processes are the basis of MetS, polyphenols, identified for their antioxidant and anti-inflammatory properties [155,156], appear as suitable candidates for preventing its development. The review of the mechanisms linking polyphenols and alleviation of these features follows.

### 4.1. Antioxidative Effects of Polyphenols

Polyphenols are known as major contributors to the fruit total antioxidant activity [157]. They do so by donation of an electron or hydrogen atom to reactive oxygen, nitrogen, and chlorine species based either on hydrogen atom transfer or single electron transfer by proton transfer [158]. Reacting with the inner side of the plasma membrane hydrophobic compounds, polyphenols impede lipid and protein oxidation, thus protecting the structure, fluidity, and function of the cell membrane [159]. Polyphenols also chelate transition metals as Fe^2+^, thus directly reducing the Fenton reaction and preventing oxidation by highly reactive hydroxyl radicals [160].

Reactive oxygen species (ROS) play leading roles in provoking pan-cellular inflammation. Their action is mediated by the activation of powerful transcription factors such as nuclear redox factor-2 (Nrf2), nuclear factor-kappa B (NF-κB), and activator protein 1 [161]. Under unstressed conditions, inactive Nrf2 is linked to its cytoplasmic regulator actin-anchored Kelch-like ECH-associating protein 1. Due to reactive species accumulation in cells under OxS, Nrf2 dissociates from Kelch-like ECH-associating protein 1, translocates into the nucleus where it modulates antioxidant-responsive elements-mediated transcription cytoprotective genes [162,163]. Phenolic compounds are able to enhance protective pathways by activating Nrf2. For example, flavonoids induce Nrf2 translocation to the nucleus where it forms heterodimers with the leucine zipper-like transcription factors small musculo-aponeurotic fibrosarcoma proteins, then bind to antioxidant-responsive elements to activate target genes such as Phase II enzymes involved in detoxification [164]. In addition, Zhang et al. [165], using human breast cells, hepatic human liver cancer cells, and mouse Hepa-1 cells, demonstrated cell-dependent agonist/antagonist effects of several flavonoids on the regulation of aryl-hydrocarbon receptor-mediated signalling pathways. Polyphenols may also suppress pro-oxidative response by down-regulating the synthesis of the pro-inflammatory interleukin-(IL)-1β, tumor necrosis factor-alpha (TNFα), and interferon-gamma. They do so by modulating NF-κB and mitogen-activated protein kinase signaling pathways [166]. Moreover, phenolic compounds can display genoprotective effects, thereby preventing/reversing the progression of various CMD diseases [167]. Through the protection of DNA against OxS damages, some types of polyphenols can promote metabolic health [168,169,170,171]. For example, epigallocatechin gallate and resveratrol exhibit high capacity to decrease DNA double strand breaks, chromosome loss, and DNA deterioration response in H_2_O_2_-induced genotoxicity in cells [172,173]. A human supplementation trial has also shown the ability of green tea polyphenolic antioxidants to increase the resistance of DNA to oxidation damages [174].

### 4.2. Anti-Inflammatory Effects of Polyphenols

The crosstalk between oxidative and inflammation signalling pathways is important for cell homeostasis and survival. Indeed, overproduction of ROS has been shown to stimulate the release of pro-inflammatory cytokines. Reciprocally, NF-κB controlled genes are able to regulate ROS production [175,176]. Various studies, particularly those oriented toward cancer, revealed that polyphenols are able to control chronic inflammatory responses at the level of cytokine production, NF-κB-mediated gene expression, and the release of the tumor-suppressing transforming growth factor-beta [177,178,179].

Furthermore, closely related to the MetS, our group has more recently demonstrated, in post-confluent Caco-2/15 cells, that polyphenols extracted from dried apple peels (DAPP), containing phenolic acids, flavonol glycosides, flavan-3-ols, and procyanidins prevented iron-ascorbate-mediated lipid peroxidation and counteracted lipopolysaccharide-mediated inflammation as shown by the down regulation of the cytokines TNFα and IL-6, lessening of cyclooxygenase-2 expression, and production of prostaglandin E_2_ via the decline of NF-κB. Concomitantly, the alleviation of inflammation was accompanied by the induction of Nrf2 (orchestrating cellular antioxidant defenses and maintaining redox homeostasis) and peroxisome proliferator-activated receptor gamma coactivator 1-alpha (the “master controller” of mitochondrial biogenesis) [180]. We later demonstrated that DAPP prevented and alleviated dextran sodium sulfate-induced intestinal inflammation in mice, stimulated antioxidant transcription factors, and improved mitochondrial dysfunction. They also decreased lipid peroxidation and up-regulated antioxidant enzymes, while decreasing the activity of myeloperoxidase, the expression of cyclooxygenase-2, and the production of prostaglandin E2. Moreover, DAPP partially restored mitochondrial redox homeostasis, fatty acid β-oxidation, ATP synthesis, apoptosis, and regulatory mitochondrial transcription factors [181]. As DAPP in parallel decreased the relative abundance of *Peptostreptococcaceae* and *Enterobacteriaceae* bacteria [182], it is possible that the modifications of intestinal microbiota brought about the amelioration of oxidative and inflammatory processes.

### 4.3. Epigenetic Control of Polyphenols

Epigenetic modification of gene expression is extremely important in the modulation of the OxS and inflammatory pathways briefly described above. Such alterations have been associated with abnormalities in various metabolic pathways [183]. Epigenetic control includes methylation of CpG rich regions, post-transcriptional modulation of chromatin histone/non-histone, and micro-RNAs (miRNAs) regulating gene expression [184,185,186]. Importantly, while these alterations may persist for cells lifespan and may have inter-generational effects, they are reversible and have thus become prime targets for interventions in the treatment of MetS [187]. Unfortunately, data on these mechanisms, particularly those bearing on DNA methylation and histone acetylation, are scarce. Table 3 shows that, in both animal models and human studies, polyphenol consumption decreases obesity, IR, and hypertension. These metabolic improvements are associated to epigenetic modifications, including increased DNA methylation, and histone methylation and acetylation.

Interestingly, reports involving miRNAs are more profuse. Table 4 summarizes the major findings obtained regarding the modulation of miRNAs and their effect on the expression of specific genes. It can be appreciated that polyphenols, independently of their origin, modulate miRNAs, especially controlling OxS and inflammation pathways in different cell models. In animal models, they also regulate miRNAs expression involved in the control of inflammation, obesity, lipogenesis, and energy expenditure. One human study shows that polyphenol consumption modulates miRNAs expression related to inflammation processes.

## 5. Polyphenol Metabolites

In the first part of the present review, we have defined the polyphenols, described their structure, reported their digestion and absorption, elaborated on their mode of action and potential benefits, emphasized their epigenetic regulation, and pointed out their interaction with intestinal microbiota. The goal of the second part is to focus on the polyphenol metabolites, derived from their colonic microbial metabolism and biotransformation. As mentioned above, parent or native polyphenols have glycosidic linkages, which limit their absorption in the small intestine, forcing them to continue their way to the colon. It is in this part of the large intestine that glycosides are cleaved and further metabolized by microbiota to potentially generate more active and better-absorbed metabolites. Using transporters and passive diffusion, the small molecular weight end-products have easy access to the circulation. Hence, the second part of this review is dedicated to specify the biological activity and consequential functional effects of polyphenol metabolites on CMD. Table 5 provides information on the different metabolites issued from colonic digestion and on their pleiotropic effects.

### 5.1. Flavonoid Metabolites and Their Antioxidant and Anti-Inflammatory Effects

Bacterial flavonoid catabolism in the colon follows a generic pattern yielding non-specific metabolites such as hydroxylated phenylpropionic acid (HPPA) and phenylacetic acids (HPAA) that ultimately may be subjected to β-oxidation and glycination to yield hippuric acid and its hydroxylated counterpart [34,217]. It can therefore be appreciated that a relatively small number of degradation products emanate from extremely diverse parent flavonoids.

#### 5.1.1. Flavonol Quercetin Metabolites

Quercetin (2-(3,4-dihydroxyphenyl)-3,5,7-trihydroxychromen-4-one) is supplied by apples, tea, red wine, berries, tomatoes, and onions. This polyphenol has attracted a lot of interest from the biomedical milieu in view of its beneficial properties in prevention-diseases (e.g., T2D, CVD) despite its limited bioavailability because of its low solubility, weak stability in the upper gastrointestinal tract, rapid fast metabolism, and short biological half-life [98]. However, quercetin molecules unable to be absorbed in the small intestine reach the colon, and are metabolized into a series of phenolic acids by luminal bacteria [265]. In fact, the health-promoting properties observed with quercetin could account for by microbiota-mediated metabolites.

Quercetin, quercetin 3-*O*-rutinoside (rutin) and their colon-derived flavonoid metabolites 3,4-dihydroxyphenylacetic acid (3,4-HPAA), 4-hydroxyphenylacetic acid (4-HPAA), 3-hydroxyphenylacetic acid (3-HPAA), and hippuric acid (*N*-benzoylglycine) have been shown to possess antioxidant and anti-inflammatory properties. On the other hand, when in vitro studies using primary cultures of rat liver parenchymal cells or the human HepG2 hepatoma cell line were treated with t-butylhydroperoxide to produce OxS, the radical scavenging properties were noted only for the parent molecules quercetin and rutin, as well as for 3,4-dihydroxytoluene (3,4-DHT). The metabolites 3-HPAA, 4-HPAA, and hippuric acid remained almost ineffective [208]. Similarly, quercetin and 3,4-DHT showed the potency to inhibit cholesterol synthesis. Another in vitro study, testing the radical scavenging capacity of 3,4-DHPAA, 3-HPAA, and 4-HPPA in human polymorphonuclear cells (stimulated with opsonized zymosan or N-formyl-methionyl-leucyl phenylalanine) revealed that out of the 3 metabolites investigated, only 3,4-DHPAA was active [209]. This radical scavenging capacity is of utmost importance when considering the cell protection from cytotoxicity. Indeed as an adjunct to its radical scavenging properties, 3,4-DHPAA activates phase 2 cytoprotective enzymes (i.e., hemeoxynegese-1, glutathione *S*-transferase), increases the gene and protein expression of aldehyde dehydrogenase isozymes in mouse hepatoma Hepa1c1c7 cells, and induces nuclear translocation of Nrf2 and aryl hydrocarbon receptor [210,211], all involved in controlling cellular REDOX status and in inhibiting cell cytotoxicity.

In vitro studies have also established the anti-inflammatory properties of quercetin metabolites. For example, experiments with lipopolysaccharide-stimulated human peripheral blood mononuclear cells showed the modulatory effect of 3,4-DHPPA, 3-HPPA, 3,4-DHPAA, 3-HPAA, and 4-hydroxybenzoic acid on the expression of the central pro-inflammatory cytokines; TNFα, IL-1β, and IL-6. TNFα expression was also significantly decreased by the metabolites 3,4-DHPPA, 3,4-DHPAA, and 4-hydroxy-hippuric acid (4-HHA), whereas 3,4-DHPPA and 3,4-DHPAA only suppressed secretion of IL-1β and IL-6 [212]. In another study, in which lipopolysaccharide was used to induce inflammation in RAW 264.7 murine macrophage cell line, 3,4-DHT disclosed an anti-inflammatory capacity by modulating the I κB/NF-κB signaling pathway [266].

Surprisingly, very little research has highlighted the potential anti-diabetic properties of colonic metabolites derived flavonoids. However, when the ability of DHPAA and HPPA was examined on β cell function, the findings clearly emphasized an elevated glucose-stimulated insulin secretion, high protection against tert-butyl hydroperoxide-induced β cell toxicity, hence better survival and function of β cells [215]. The last functions were mediated by the activation of protein kinase C and the extracellular regulated kinases (ERKs) pathways. Collectively, these interesting data suggest that flavonol quercetin/rutin metabolites exert anti-diabetic actions.

#### 5.1.2. Flavones and Flavanones Metabolites

Flavones (e.g., apigenin) are present in foods as cereals, parsley, thyme, celery, and citrus fruits under their *O*-glycosidic or C-glycosides derivatives. Once glucosides are hydrolyzed at the intestinal level by digestive enzymes, unabsorbed aglycons undergo further reactions in the large intestine by specific micro-organisms (*Clostridium orbiscindens*, *Enterococcus avium*) causing C-ring fission. Following the metabolism of apigenin, metabolites [e.g., phloretin chalcon, 3-(4-hydroxyphenyl)-propionic, 3-(3-hydroxyphenyl)-propionic, and 4-hydroxycinnamic acids] are acquired [221].

Flavanones (e.g., naringenin) are abundant in tomatoes and citrus fruits, and seem to have higher bioavailability in comparison to flavonols and flavan-3-ols [267]. Flavanone deglycosylation and further degradation by colonic microbiota pathways follow the similar fate of flavonols [267]. *Clostridium* species and *Eubacterium ramulus* are largely involved in these transformations in the colon [217,268]. Metabolites, including 3-(3,4-dihydroxyphenyl)-propionic, 3-(4-hydroxyphenyl)-propionic, and 3-(3-hydroxyphenyl)-propionic acids are produced following naringenin metabolism. However, as for quercetin metabolites, hydroxyphenyl propionic acids (derived from apigenin and naringenin metabolism) display antioxidant and anti-inflammatory capacities (Table 5).

#### 5.1.3. Isoflavone Daidzein and Daidzin Metabolites

Isoflavone, as a phytoestrogen precursor, is another flavonoid class of interest. Like many types of polyphenols, isoflavones exert advantageous effects on intestinal health, menopause symptoms, hormone-mediated syndromes, and CVD [269]. They are also transformed into their active metabolites by enzymes from the gut microbiota to generate compounds endowed with elevated estrogenic activity (e.g., 4′,7-isoflavandiol; equol) or inactive compounds (e.g., *O*-desmethylangolensin; *O*-DMA) [270]. Importantly, the majority of humans are competent to yield *O*-DMA, but only 25–50% among them engender equol [271,272].

The potential health properties of equol and *O*-DMA, both derived from daidzein and its 7-*O*-glycoside daidzin, are the subject of continuing debate. Some studies claim their beneficial effects on the risk of coronary heart disease events [273,274] and others have shown a limited action [275]. The diverging conclusions stems in part from the inter-individual variability in the gut microbiota [276] or from a characteristic that is influenced by ethnicity [277]. Interestingly enough, prehypertensive postmenopausal women producing equol/*O*-DMA had more favorable cardiovascular risk profiles than non-producers [278]. Furthermore, equol/*O*-DMA producers exhibited lower serum uric acid, triglycerides, and waist/hip ratio, as well as a tendency to have higher high density lipoprotein-cholesterol compared to equol non-producers [279]. These effects are probably exerted through the control of cell redox potential as both equol and *O*-DMA have antioxidant capacity, exemplified by the stimulation of catalase and total superoxide dismutase activities [226]. In a population-based, cross-sectional investigation, urinary concentrations of isoflavones and equol/*O-*DMA were related to well-disposed insulin sensitivity and cardiometabolic markers in pregnant women [280]. Other studies emphasized the beneficial role of *O*-DMA in obesity [281]. However, caution should be exerted when concluding on the effects of isoflavone and related metabolite interventions, as sufficiently randomized controlled trials with statistical are needed [275].

#### 5.1.4. Flavanol Catechin Metabolites

Flavanols or flavan-3-ols are among the most consumed phenolic compounds in Western populations [282]. They are composed of monomers (e.g., catechin, epicatechin, epigallocatechin) and oligomers/polymers (known as proanthocyanidins) [283]. Several groups have reported their ability to prevent chronic diseases (e.g., T2D and CVD) [283,284,285]. Hydroxy-phenyl-γ-valerolactones and their related hydroxy-phenylvaleric acids are the main metabolites of dietary flavan-3-ols [87,286]. Although a number of studies based on different models aiming at assessing hydroxy-phenyl-γ-valerolactones bioactivity have been reported in the past decade, those involving human subjects are scarce. Inflammatory and OxS processes were particularly studied. For example, (3′4′-dihydroxyphenyl)-γ-valerolactone significantly decreased TNFα-induced NF-kB transcriptional activity in HepG2 cells [92]. Furthermore, a decrease was noted in nitrous oxide production and inducible nitrous oxide synthase expression in the murine macrophage RAW264.7 cells and freshly isolated human monocytes, both treated with (δ-(3,4-dihydroxyphenyl)-γ-valerolactone) a metabolite of the maritime pine bark Pycnogenol [233].

The accumulation of pro-inflammatory cytokine-producing T-lymphocytes and macrophages with the concomitant secretion of the vascular cell-adhesion molecule-1 by the endothelium is key to the early development of the atherosclerotic plaque [287]. Inhibition of this process was observed following the incubation of cultured human umbilical vein endothelial cells with 3′,4′-dihydroxyphenyl)- γ-valerolactone [234], thereby providing the evidence that this compound is a potential therapeutic target. Transferability of the concentrations (quantities) of natural compounds to in vivo or clinical environment (cytotoxicity) represents a major limit of these in vitro experiments. The polyphenolic metabolites 3′,4′,5′-trihydroxyphenyl)-γ-valerolactone and 5-(3′,5′-dihydroxyphenyl)-γ-valerolactone also displayed, in spontaneously hypertensive rats, the capacity to lower angiotensin-1 converting enzyme activity and blood pressure, both contributing to improving endothelial function and arterial elasticity [235]. Paradoxically, the flavan-3-ol metabolites 5-(3′,4′,5′-trihydroxyphenyl)-γ-valerolactone, 5-(3′,4′-dihydroxyphenyl)-γ-valerolactone, and 5-(3′-methoxy, 4′-hydroxyphenyl)-γ-valerolactone did not improve vascular endothelial plasticity of freshly isolated mouse saphenous arteries [236]. The dissimilarity in data could partially be explained by the different models used, especially that isolated saphenous artery endothelium is less reactive than the vessels of the spontaneously hypertensive rat. The in vivo versus the in vitro situations and the experimental time frame are also factors that have to be considered.

The decrease in obesity, a major element in the development of MetS, in response to the consumption of foods rich in flavan-3-ols (e.g., green tea), has been attributed to the role of their content on polyphenolic metabolites in increasing energy expenditure via the modulation of cell differentiation and thermogenesis in adipose tissues [231]. However, when tested in vitro, the flavan-3-ol metabolites [5-(3’,4’-dihydroxyphenyl)-γ-valerolactone, 5-(3’-hydroxyphenyl)-γ-valerolactone-4’-O-sulfate, and 5-phenyl-γ-valerolactone-3’,4’-di-O-sulfate] did not stimulate cell differentiation and the activation of immortalized pre-adipocytes. In addition, none of the metabolites regulated the expression of the uncouple protein 1, nor the main transcription factors implicated in brown adipocyte genesis. Nevertheless, both 5-(3’,4’-dihydroxyphenyl)-γ-valerolactone and 5-(3’-hydroxyphenyl)-γ-valerolactone-4’-O-sulfate protected immortalized pre-adipocytes from H_2_O_2_ generated OxS [231]. In a recent clinical trial, Rodriguez-Mateos et al. [288] showed that cranberry juice given to healthy volunteers improved, in a concentration- and time-dependent manner, the flow-mediated vasodilatation, an early clinical sign in the development of atherosclerosis. Interestingly, the plasma level of the flavan-3-ols metabolite, 5-(3′-hydroxyphenyl)-γ-valerolactone-4′-sulfate, was significantly positively correlated to flow-mediated vasodilatation. Noteworthy, mice on high-fat diet improved non-alcoholic fatty liver disease by lessening IR and endotoxin-toll-like receptor 4/NF-*κ*B pathway in response to raised hepatic catechin metabolites (namely phenyl-*γ*-valerolactones) following catechin-rich green tea extract [289].

Overall, these findings offer promising beneficial health effects of polyphenols metabolites. However, additional clinical studies are necessary to validate the data in humans and to ensure their transferability from in vitro and animal models to in vivo human situations.

#### 5.1.5. Tannin Ellagitannin and Urolithin Metabolites

Hydrolysable tannins, named ellagitannins, encompass one or more gallic acid units and one or more hexahydroxydiphenoic acid units, ester-connected with a sugar residue. In view of their difficulty to be absorbed in the small intestine, ellagitannins reach the colon where they are hydrolyzed into mainly ellagic acid and urolithin (3,4-benzocoumarin derivatives). Supporting data propose a stringent relationship between the consumption of ellagitannins-rich foods and healthy effects based on animal and human models.

The rat under high carbohydrate high-fat diet-induced MetS model has demonstrated the ability of ellagic acid to improve hepatic and cardiometabolic functions, while normalizing glucose tolerance, blood lipid components, central obesity, and body weight [290]. The mechanisms involved in the modulation of OxS and inflammation are through the protein level regulation of Nrf2 and NF-κB, with an impact on metabolic pathways such as fatty acid β-oxidation [290]. Other studies confirm the health benefit properties of ellagic acid, for example, by showing that it dose-dependently repressed the IL-1β-induced production of ROS [248]. This was associated with a significant reduction of the attachment of the established human monocyte cell line to IL-1β-treated human umbilical vein endothelial cells through the suppression of NF-κB, thereby downregulating the expression of vascular cell-adhesion molecule-1 and E-selectin and resulting in decreased monocyte adhesion. Along the same line of evidence, ellagic acid significantly inhibited oxidized low-density lipoprotein and platelet-derived growth factor-BB-induced proliferation of rat aortic smooth muscle cells via inactivation of ERK and marked decrease of proliferating cell nuclear antigen. [249]. More evidence for ellagic acid effects in preventing the development of atherosclerotic events has been provided by showing its capacity of reducing platelet growth factor (PDGF) receptor-β-phosphorylation, diminishing ROS production, and lessening downstream activation of ERK, and finally blocking platelet-derived growth factor-induced expression of cyclin D1 in primary cultures of rat aortic smooth muscle cells [291]. Moreover, these in vitro results were further supported by the ability of ellagic acid to block T2D-induced medial thickness, and lipid and collagen deposition in the arch of aorta in streptozotocin-induced diabetic rats [291]. Collectively, these data suggest that ellagic acid can inhibit OxS and inflammation response, which thus favorably contributes to the regulation of vascular homeostasis.

Urolithins, formed primarily from the oxidative linkage of galloyl groups, have extensively emerged as novel natural bioactive compounds, being currently the focus of extensive investigations. The type of urolithin produced depends on the colon microbiome. Three ellagitannins-metabolizing metabotypes have been identified: ‘metabotype A’ (producers of the dihydoxylated urolithin-A conjugates), ‘metabotype B’ (producers of urolithin-A/isourolithin-A conjugates and/or monohydoxylated urolithin-B conjugates), and ‘metabotype 0′ (no urolithins produced) [292]. Contrary to the parent compounds, which bloodstream concentrations are negligible, these ellagic acid derivatives may reach micromolar concentrations and thus act as surrogate biomarkers of the inter-individual variation in the colon metabotype [245]. Among the numerous metabolites, urolithin-A has been particularly studied in terms of its anti-inflammatory and anti-oxidative properties both in vitro and in vivo [250,293,294], as well as anti-atherosclerotic and anti-angiogenic activities [293,295]. In line with these features, adhesion model involving established human aortic endothelial cells and human leukemia monocytic THP-1 cells showed that urolithin-A inhibited TNFα-stimulated adhesiveness and endothelial cell migration [257]. To delve deeper and uncover the mechanisms, the authors could further demonstrate that these effects were concomitant to a down regulation of the levels of chemokine ligand 2, plasminogen activator inhibitor-1, and IL-8. Even more interesting, a clinical trial provided evidence that overweight-obese individuals consuming ellagitannin-containing food with the urolithin metabotype producing urolithin-A were protected against CMD when compared to those with a urolithin-B metabotype [296]. Despite the small number of participants, this clinical trial pointed out the important inter-individual disparity in metabolizing polyphenols, the regulation by microbiota, and indicated that urine metabolite profiling is a valuable tool in assessing potential effects. Fecal polyphenol metabolite profiling could also shed some light on the mechanisms of action. In this context, the profile of fecal phenolic-derived metabolites correlated to blood biomarkers of OxS and inflammation, thus providing support to this avenue [297].

Urolithin may constitute a central regulator of CMD as IR decreased in response to urolithin administration to mice fed high-fat, high-sucrose diet [298]. Concomitantly, urolithin lowered glucose, fatty acids, and triglycerides, while ameliorating adiponectin and mitochondrial function. Furthermore, in the same animal model, through thyroid hormone modulation, urolithin increased energy expenditure by enhancing thermogenesis in brown adipose tissue and inducing browning of white adipose tissue [299]. Therefore, this first-in-class natural compound contributed to metabolic homeostasis via its capacity to normalize OxS, inflammation, obesity, and IR, which overall contribute to MetS.

### 5.2. Non-Flavonoids

#### 5.2.1. Lignan Metabolites

Plant lignans are non-flavonoids, but polyphenolic, phytoestrogenic compounds. They exhibit several biological functions with potent protective benefits. Anti-estrogenic, antioxidant, anti-inflammatory, anti-atherosclerotic, and anti-carcinogenic effects are among their properties, thereby conferring therapeutic values [300,301]. These properties explain the impact of lignan-rich diets on the prevention of hyperlipidemia, IR and hyperglycemia, T2D, and CMD [302,303,304]. The most frequent forms of dietary lignans are secoisolariciresinol, lariciresinol, pinoresinol, and matairesinol, which are metabolized by gut microflora enzymes into enterolignans such as enterodiol and enterolactone undergoing enterohepatic circulation [305,306]. At least, 28 bacterial species belonging to 12 different genera (e.g., *Bacteroides*, *Clostridium*, *Bifidobacterium*, and *Ruminococcus*) have been identified to be involved in the transformation of lignans to enterolignans [307]. Several groups have quantified the concentrations of the enterolactone metabolite in blood and urine, and reported a protective association in view of the reduction (30–45%) of cardiovascular mortality [308]. Similarly, in a prospective investigation, enterolactone (more than enterodiol) was associated with a lower risk of T2D in U.S. women [309]. A cross sectional trial of the general U.S. population found that subjects with high excretion of enteractone had lower odds of having MetS [310]. Moreover, in a representative sample of U.S. adults, the cross-sectional analysis of data detected that urinary enterolignan concentrations are associated with lower serum triglyceride and greater high-density lipoprotein-cholesterol concentrations in U.S. adults [311]. Additional efforts are obviously needed to confirm these interesting cardiometabolic findings and to explore potential mechanisms of action behind diverse enterolignan metabolites.

#### 5.2.2. Resveratrol Metabolites

Resveratrol (3,5,4′-trihydroxystilbene) belongs to the stilbene derivative and is a dietary polyphenolic compound accessible in 70 plant species such as grapes, mulberries, and peanuts [312]. Its pleiotropic effects have made it so famous that it is called a star molecule. It displays numerous biological activities, including antioxidant, anti-inflammatory, cardioprotective, anti-aging, and cancer-chemopreventive features [313,314]. Accordingly, the wealth of scientific reports revealed the effectiveness and beneficial actions of resveratrol in MetS and related disorders such as obesity, IR, and T2D [315,316]). For example, a meta-analysis evaluating 11 studies involving 388 T2D subjects mentioned that resveratrol markedly lessened fasting glucose, insulin, haemoglobin A1c, and IR [278]. Resveratrol was also able to improve hypertension by acting on vascular nitric oxide production, reducing endothelial dysfunction and arteriolar remodeling [317,318]. However, other studies could not record advantageous data on MetS in response to resveratrol [319].

Today, it is well established that resveratrol is easily absorbed by the intestine and promptly metabolized to produce glucuronide and sulfate conjugates [320,321]. Although many gaps are still persistent in the metabolic route of resveratrol, intestinal bacteria transform resveratrol into dihydroresveratrol, known to be partially absorbed and subsequently metabolized to conjugated forms, which can be excreted in urine [322,323]. Besides dihydroresveratrol, two additional bacterial *trans*-resveratrol metabolites were identified: 3,4′-dihydroxy-*trans*-stilbene and 3,4′-dihydroxybibenzyl (lunularin) [264]. Large inter-individual variation was noticed in different subjects: some of them were lunularin producers, others were dihydroresveratrol producers, and the last category was mixed producers, according to levels of these metabolites [264]. Clearly, there was a close relationship between lunularin producers and the abundance of *Bacteroidetes*, *Actinobacteria*, *Verrucomicrobia*, and *Cyanobacteria*, along with lower abundance of *Firmicutes* compared to dihydroresveratrol or mixed producers [264]. Whether resveratrol metabolites are physiologically relevant to tackle metabolic disorders still remains an enigma.

## 6. Conclusions

Consuming a diet rich in fruits and vegetables is associated with a reduced risk of several noncommunicable lifestyle-related diseases, including CVD. They contribute several essential and bioactive micronutrients such as polyphenols. The first goal of this review was to focus on the available and pertinent literature, which assessed the health-promoting potential of polyphenols to modulate OxS and inflammation. These two major components are powerful inducers of IR, leading to CMD, especially MetS and related complications such as T2D and atherosclerosis.

For the reader’s benefit, our review first summarized the most widely distributed classes, sources, and properties of the naturally occurring polyphenols, separated into two main groups, flavonoids and non-flavonoid compounds. Second, attention was paid to their transformation in the intestinal lumen, absorption in different regions of the GI tract, and their bioavailability in the bloodstream. Clearly, most of polyphenols undergo complex intraluminal transformation during digestive and absorptive processes. Data obtained from critical literature revision underline the poor bioavailability, aqueous solubility, permeability, and instability of especially polyphenols with high molecular weights. Third, considerable emphasis was devoted to the essential polyphenol-microbiota interaction as polyphenols directly exert their regulatory effects on intestinal bacteria with concomitant biotransformation of polyphenols into metabolites by gut microbiota. This fundamental interplay provides the central explanation as to the stupefying pleiotropic biological/clinical actions and functions of “parent’’ polyphenols whereas their systemic bioavailability is incredibly limited, which highlights the prominent contribution of polyphenol metabolites. Fourth, the multifaceted actions and functions of ‘’parent’’ polyphenols were summed up. Not only do polyphenols show the great ability to regulate cellular, molecular, and physiological pathways in relation with OxS and inflammation, but they also exhibit a remarkable effectiveness in improving cardiometabolic biomarkers to the advantage of human health. Thanks to their anti-obesogenic, -hyperlipidemic, -hypertensive, -IR, -atherosclerotic, as well as -diabetic effects, they may help to prevent and treat CMD as global public health problems with vast mortality and economic consequences. Fifth, although polyphenol metabolites were also shown to play a similar beneficial role in metabolic complications, there are various enigmatic issues to deal with: inter-individual differences in the microbial conversion of parent polyphenols, largely unknown microbial metabolites from distinct polyphenols, unidentifiable bacterial strains involved, metabolite health-promoting effects of a multitude of metabolites, elusive metabolite doses, the underlying mechanisms of their specific actions, and the lack of clinical trials.

## Figures and Tables

**Table 1:** Gastric handling of polyphenols.

Substrates	Sources	Experimental Conditions and Model	End Products	Main Observations	Conclusions	Ref
Procyanidins polymers (2–6 mers)	Cocoa	Incubation in simulated gastric juice (pH 2.0) at 37 °C for up to 3.5 h	Catechin/epicatechin Monomer and dimer	Time-dependant hydrolysis of oligomers.	Role of stomach in the processing of phenolic compounds.	[64]
Dimeric catechin/ epicatechin	Cocoa	Incubation in simulated gastric juice (pH 1.8) at 37 °C for up to 60 min	Catechin/epicatechin Dimers isomerization	Time-dependant hydrolysis of dimers.	Role of stomach in the processing of phenolic compounds.	[65]
Free and conjugated Hydroxytyrosol and Tyrosol	Olive oil	Incubation in simulated gastric juice (pH 2.0) at 37 °C for up to 4 h	Free hydroxytyrosol and tyrosol	Time-dependent hydrolysis of hydroxytyrosol and tyrosol conjugates.	Stomach hydrolyzes phenolic compounds conjugates.	[66]
Monomeric/ Polymeric Catechin/ epicatechin	Grape seed extract	Incubation in simulated gastric juice (pH 2.0) + pepsin at 37 °C for 2 h	Catechin/epicatechin Oligomers	Stability of catechin/epicat.	Stability of phenolic compounds at the gastric level.	[67]
Hydroxycinnamic acid derivatives, Flavonols, dihydrochlcones monomeric flavans-3-ols, procyanidin B_2_	Apple juice	Incubation in simulated gastric juice (pH 2.0) + pepsin at 37 °C for up to 4 h	Hydroxycinnamic acid derivatives, flavonols, dihydrochlcones monomeric, Epicatechin monomer	Stability of Hydroxycinnamic acid derivatives, flavonols, dihydrochalcones monomeric. Hydrolysis of procyanidin B_2_.	Stability of phenolic compounds in the stomach dependent on structure.	[68]
Purified Hesperidin 2S	*Citrus sinsensis* peel extract	Digestion in the simulator of human intestinal microbial ecosystem (pH 2.0).	Intact hesperidin 2S	No degradation of Hesperidin 2S.	Hesperidin is resistant to the degradation in the stomach.	[69]
Resveratrol caprylic esters	Synthesis product	Incubation in simulated gastric juice (pH 1.2) + pepsin at 37 °C for up to 2 h.	Intact resveratrol caprylic esters	No hydrolysis of resveratrol caprylic esters.	Resveratrol caprylic esters are not metabolized in the gastric phase.	[70]
Polyphenols	Simulated oral digestion of peeled apple tissue	Incubation in simulated gastric juice (pH 1.6) + pepsin at 37 °C for up to 1 h in a dynamic rat stomach wall model	Released from initial material in deceasing order: chlorogenic acid, epicatechin, catechin, procyanidin B_2_ flavan-3-ols, hydoxycinnamic acids, dihydrochalcones flavonols.	All polyphenols were stable except for procyanidin B_2_ that was hydrolyzed to epicatechin.	Polyphenol resistance to degradation dependent on structure.	[71]
Polyphenols	Simulated oral digestion of Kiwifruit tissue	Incubation in simulated gastric juice (pH 1.2) + pepsin at 37 °C for 2 h	Release of the 16 identified polyphenols from initial material during stomach digestion: catechin, epicatechin, quercetin, rutin, chlorogenic acid, caffeic acid, ferulic acid, p-coumaric acid, gallic acid, salicylic acid, vanillic acid.	All polyphenols were stable.	Polyphenol resistance to degradation.	[72]
Procyanidins oligomers and flavonol monomers	Cocoa	Oral administration of procyanidin oligomers and flavonol monomers to healthy subjects. Gastric contents collected and analyzed at 20 min.	Intact procyanidins oligomers and flavonol monomers	Stomach Procyanidins oligomer and flavonol monomer profiles similar to original product.	Procyanidins oligomers and flavonol monomers are stable in the gastric environment.	[73]
Caffeic acid, gallic acid, chlorogenic acid, ferulic acid, coumaric acid	Purchased purified phenolic acids	In vivo rat ligated pylorus model for in situ gastric digestion at 37 °C for 25 min. Portal vein and abdominal aorta blood collected. Plasma analyzed with and without sulfatase and β-glucuronidase treatment.	Intact and conjugated coumaric acid, ferulic acid, caffeic acid, gallic acid, chlorogenic acid	Rapid appearance of coumaric acid > ferulic acid > caffeic acid > gallic acid > chlorogenic acid in portal vein and abdominal artery. Rapid appearance mainly of coumaric and ferulic acid conjugates in portal vein and abdominal artery.	Differential absorption efficiency of phenolic acids and differential affinity of monocarboxylic acid transporters.	[74]
Flavone glycosides (apigenin, luteolin, chrysoeriol) and flavonoid glycosides (kaempferol, quercetin, isorhamnetin)	Parsley	Oral administration of glycoside extracts to rats. Animals sacrificed at 1, 1.5, 2, 4, or 12 h post administration. GI tract segmented (stomach, small intestine, colon, cecum). Stomach wall and lumen content analyzed at 2 h.	At 2 h: flavonoid glycosides in the stomach lumen and wall. Quercetin aglycone in the stomach wall.	At 2 h: flavonoid composition of stomach wall similar to stomach lumen but concentration lower. One aglycone present.	Stomach absorbs intact flavonoid glycosides.	[75]
Isoflavones (Daidzein, daidzin, genistein, and genistin)	Commercial source	In vivo rat ligated pylorus model for in situ gastric digestion at 37 °C for 25 min. Jugular vein blood analyzed for daidzein, daidzin, genistein, and genistin up to 30 min.	Daidzein and genetein (isoflavone aglycones)	Time-dependent absorption and transport of Daidzein and genetein, but not their respective glycosides daidzin and genistin.	Selective absorption and transport of isoflavone aglycones by the stomach.	[76]
Quercetin, rutin, and isoquercetin	Commercial source	In vivo rat ligated pylorus model for in situ gastric digestion at 37 °C for 30 min. Biliary duct cannulation and content analyzed. Aortic blood collected and analyzed.	Biliary quercetin and 3′-O-methyl-quercetin	Querecetin absorbed by the stomach and secreted in bile. No absorption of rutin or isoquercetin.	Limited role of the stomach in flavonoid glycosides. Selective absorption and transport of aglycones.	[77]

**Table 2:** Experimented Caco-2 cells in polyphenol transport and metabolism.

Substrates	Sources	Experimental Conditions	Main Observations	Conclusions	Ref
hydroxytyrosol, tyrosol, and oleuropein	Olive oil	Polyphenols added to the apical chamber and incubation for 2 h. Apical and basolateral compartments collected and analyzed.	↓ hydroxytyrosol and tyrosol in the apical and ↑ in the basolateral media. Appearance of 3-*O*-methyl-tyrosol and glutathionyl-hydroxytyrosol in the apical and basolateral compartments. Φ transport of oleuropein from the apical to the basolateral compartment.	Hydroxytyrosol transported through the enterocyte apical membrane to the basolateral compartment with formation of conjugates.	[66]
*Trans*-piceid (Resveratrol 3-β-mono-d-glucoside)	*V. Vinifera*	Incubation with trans-piceid up to 360 min.	Bidirectional (apical to basolateral and inverse) transport of trans-piceid. Detectable trans-resveratrol in both chambers.	Trans-piceid and its aglycone are transported across the apical side and effluxed by the basolateral membrane.	[82]
*Trans*-piceid (Resveratrol 3-β-mono-d-glucoside)	*V. Vinifera*	Pre-incubated with ± chrysin (5,7-Dihydroxyflavone) and ± d-saccharic lactone in 6-well plates. Incubated with *trans*-piceid or *trans*- resveratrol 24 h.	Appearance of *trans*- Resveratrol after incubation with *trans*-piceid. ↑ *trans*- resveratrol-glucuronides production in chrysin (UDP-glucuronosyl transferase inducer) treated cells.	*Trans*-piceid undergoes hydrolysis to its aglycone and *trans*-resveratrol undergoes phase II metabolism within the enterocyte.	[82]
Quercetin, Quercetin-4-*O*-β-d-glucoside, Quercetin-3-*O*-β-d-glucoside, Quercetin-3,4-di-*O*-β-d-glucoside	Purified polyphenols	Polyphenols added to the apical chamber and incubation up to 2 h. Apical, cellular, and basolateral compartments collected.	Time-dependent ↓ quercetin in apical chamber. Stability of quercetin glycoside in apical chamber. Time-dependent appearance of quercetin glycosides in apical chamber and cellular compartment when quercetin is added. Time-dependent ↑ quercetin glycosides in basolateral compartment and stable low quercetin.	Quercetin aglycone is preferentially transported through the enterocyte apical membrane to the basolateral compartment after intracellular conjugation.	[84]
Catechin, epigallocatechin gallate encapsulated or not in non-ionic surfactant-based vesicles (niosomes)	Purified polyphenols	Polyphenols added to the apical chamber and incubation up to 6 h at 37 °C or 4 °C. Cell and basolateral compartments collected and analyzed.	Time-, concentration-, and temperature-dependent uptake of polyphenols. ↑ uptake when inserted into niosomes. Time-, concentration-, and temperature-dependent transport of polyphenol to the basolateral compartment, ↑ with niosomes. ↓ transport with ATP inhibitor, ↑transport with EDTA and P-glycoproteins and multidrug resistance proteins inhibitors.	Temperature dependence of uptake suggested energy-driven process. Deactivation of efflux pumps resulted in increased uptake by apical membrane and efflux in basolateral compartment.	[86]
Free and methyl esters of Hydroxycinnamic acids (ferulic, sinapic, *p*-coumaric and caffeic methyl esters), Ethyl esters of 5,5-diferulate, 8-*O*-4-diferulate, 8,5-benzofuran	Purified polyphenols	Medium collected after 24 h incubation and analyzed.	Glucuronides of ferulic, sinapic, *p*-coumaric and caffeic methyl esters, sulfates of ferulic, sinapic, *p*-coumaric and caffeic methyl esters, ferulic, sinapic, *p*-coumaric sulfates.	Metabolites produced either intra-cellularly and excreted in medium or produced in the medium by secreted phase I and phase II enzymes.	[90]
Quercetin, Quercetin-3,7,3,4-*O*-tetra-ethylacetate	Purified polyphenols	Polyphenols added to the apical (A) or basolateral (B) chamber and incubate up to 2 h. Apical, and basolateral compartments collected.	Time- and temperature-dependent bidirectional but preferential B-A transport of quercetin and ethylacetate derivative. Transport more efficient for the ethylacetate derivative. P-glycoproteins and multidrug resistance proteins inhibitors. ↑ quercetin permeation coefficient from A-B and ↓ from B-A. Φ on ethylacetate derivative.	Quercetin might be a substrate of P-glycoproteins and multidrug resistance proteins, causing the ↓ bioavailability of quercetin. Quercetin ethylacetate derivative exhibited better membrane permeation than parent compound.	[91]
Catechin, puerarin (Daidzein-8-*C*-glucoside)	Purified polyphenols	Incubation up to 2 h.	Time- and concentration-dependent uptake. Catechin enhanced uptake and transcellular transport of puerarin but puerarin inhibited that of catechin. P-glycoproteins and multidrug resistance proteins inhibitors. ↑ polyphenol uptake and transport.	Deactivation of efflux pumps resulted in increased uptake by apical membrane and efflux in basolateral compartment.	[92]

↑: increase; ↓: decrease; Φ: no effect.

**Table 3:** Effect of polyphenols on DNA and histone methylation and acetylation.

Polyphenols	Experimental Model	Tx Duration (Week)	Polyphenol Dosage	Epigenetic Modifications	Outcomes in Response to Polyphenols	Ref
Obesity and Insulin Resistance						
Raspberry extract	HFD-fed mice	16	120 mg/kg/d	↑AT Histone methylation and acetylation	↓Obesity ↓IR ↓Inflammation ↓Liver steatosis	[188]
Quercetin and Q2 derivative	HFD-fed rats	12	0.26 mg/kg/d	↑AT Histone methylation	↓Obesity ↓IR ↓Dyslipidemia ↓Liver steatosis	[189]
Apples	HFHSD-fed rats	8	700 mg/kg/d	↑Methylation Aqp7 ↑PGC genes ↑Methylation leptin gene	↓Obesity ↓IR ↑AT lipolysis	[190]
Hypertension						
Cocoa	Humans Pre-hypertension or hypercholesterolemia	2	6 g/d	↓Leuk DNA methylation ↓ DNA Mtases methylation	ND	[191]
Resveratrol	Salt-sensitive hypertensive rats	0–12	50 g/L drinking water	↑histone H3K27me3 in renal aorta	Prevention of hypertension ↑Antioxidant defence	[192]

Tx: treatment; HFD: high-fat diet; HFHSD: high-fat high-sucrose diet; miR: micro-RNA; IR: insulin resistance; AT: adipose tissue; Leuk: leukocytes; Mtases: methylases; ND: no data; ↑: increase; ↓: decrease.

**Table 4:** Effects of polyphenols on the regulation of miRNAs on oxidative and inflammation pathways.

Polyphenols	Experimental Model/Conditions	Regulated miRNAs	Expression Pattern and Function	Ref
Cellular models				
Quercetin and Isorhamnetin	Pre LPS Tx stimulation of murine RAW 264.7 macrophages. Polyphenols (0, 10–100 µmol/L)	↓miR-155	↓TNFα, ↓ iNOS, ↓IL-1β, ↓IL-6, ↓MIP1α, ↓NF-kB ↑Nrf2 and ↑ARE	[193]
Resveratrol	Human THP-1 cell line HPBMC	↑miR-663 ↓miR-155	↓basal AP-1 and ↓LPS-induced AP-1 ↓ JunB/D mRNA	[194]
EGCG	IL-1β-stimulated human OA chondrocytes	↑hsa-miR-199a-3p	↓COX-2 mRNA/protein expression ↓PGE_2_ production	[195]
EVOO oleocanthal (OC) and oleacein (OA) secoiridoids	SGBS adipocytes pretreated with OC or OA before stimulation by TNFα	↓miR-155-5p, ↓miR-34a-5p ↑let-7c-5p	↓IL-1β, ↓COX-2, ↓MMP-2, ↓NF-kB, ↓NADPH oxidase ↑SOD and ↑GPx, ↑PPARγ ↓MCP-1, ↓CXCL-10, ↓M-CSF	[196]
Olive oil hydroxytyrosol (HT)	SGBS adipocytes pretreated with HT before stimulation by TNFα	↓miR-155-5p, ↓miR-34a-5p ↑let-7c-5p	↓MCP-1, ↓CXCL-10, ↓IL-1β, ↓IL-6, ↓vEGF, ↓COX-2, ↓M-CSF, ↓MMP-2, ↓NF-B and ↓ROS production ↑GPX ↑eNOS, ↑PGC-1α	[197]
Propolis extracts	HaCat cell line treated for 24 h with propolis extracts (3.125, 1.56, and 0.78 mg/mL)	↑miR-19a-3p ↑ miR-203a-3p ↑miR-27a-3p ↓miR-17-3p	↓TNFα mRNA ↓NFE2L2 mRNA ↑GPX2, ↑MnSOD and ↑TRXR2 mRNAs	[198]
Curcumin polyphenolic compound	ARPE-19 cells treated with 20 μΜ curcumin and 200 μΜ H_2_O_2_	↑miR-146a ↑miR-155 ↓miR-23b ↓miR-27b ↓miR-26b ↓miR-15b ↓miR-9 ↓miR-30b, miR-30e	↓NF-κB ↑CAT, ↑GPx	[199]
Açai and red muscadine grape polyphenols	HUVEC ROS induction by 25 mM glucose for 30 min	↑miR-126 ↑MiR146a	↓IL-6, ↓IL-8, ↓NF-kB, ↓PXR, ↓VCAM-1 ↑CYP1A1, ↑MDRP1, ↑CAT, ↑GST activity	[200]
Resveratrol	LPS-stimulated THP-1 macrophages pretreated with resveratrol	↑miR-Let7A	↓TNFα, ↓IL-6, ↓IL-10, ↓IL-4, ↓SIRT1 mRNAs	[201]
Animal models				
Quercetin	Ctrl or HFD C57BL/6 J fed mice 0.2 or 2.0 mg/g diet	↑miR-125b	↓IL-6, ↓CRP, ↓MCP-1, ↓AOAH, ↓HO-1, ↓Ref-1, ↓TLR-2 mRNAs	[202]
Grape seed extract	HFD-fed obese Rats 30 mg/kg/d	↓miR-33a, ↓miR-122	↓TC, ↓TAG, ↓LDL-C, ↓TNFα, ↓liver MDA ↑SOD, CAT; ↑liver GSH	[203]
Polydatin (3,4’,5-trihydroxy-stilbene-3-β-d-glucoside)	Crtl, or HFrD, HFrD+Polydatin (7.5, 15, 30 mg/kg), HFrD + PG (4 mg/kg) fed SD rats IG saline, polydatin or PG 7 week	↓ miR200-a	↓ TXNIP, ↓NLRP3, ↓ASC, ↓Casp-1, ↓ SREBP-1 and ↓SCD-1 ↑ PPAR-α and ↑CPT-1	[204]
Tea extract	HFD-fed mice for 12 weeks 500 mg/kg/d	↓miR-335 ↓ miR-155 in AT	↓Obesity, ↓IR, ↓Inflammation, ↑Energy expenditure	[205]
Resveratrol	HFHS-fed rats for 6 weeks 30 mg/kg/d	↑miR-211-3p ↑miR-1224 ↑miR-539-5p ↓AT miR-511-3p	↓Obesity, ↓AT lipogenesis	[206]
Human studies				
Resveratrol	T2D HT patients. 1-year daily intake of grape extract (8.1 mg/d for first 6 months and 16.2 mg/d for last 6 months)	HPBMC ↑miR-21 ↑miR-181b ↑miR-663 ↑miR-30c2	↓IL-6, CCL-3, IL-1β, TNFα, ↑LRRFIP-1	[207]

Tx: Treatment; Ctrl: control, HFD: high-fat diet; HFrD: high-fructose diet; PG: pioglitazone; SD: Sprague-Dawley rat; ARPE-19 cells: adult retinal pigment epithelial cell line-19; HUVEC: human umbilical vascular endothelial cells; THP-1: human monocyte leukemia cells; LPS: lipopolysaccharides; TNFα: tumor necrosis factor alpha; iNOS: inducible nitric oxide synthase; IL-1β: interleukin-1β; IL-6: interleukin-6; MIP-1α: macrophage inflammatory protein-1α; NF-kB: nuclear factor κB; Nrf2: NF-E2-related factor 2; AOAH: acyloxyacyl hydrolase; HO-1: heme oxygenase-1; Ref-1: redox factor-1; TLR-2: toll like receptor-2; ARE: antioxidant response element; CRP: C-reactive protein; MCP-1: monocyte chemo-attractant protein-1; T2D: type-2 diabetes; HT: hypertensive; HPBMCs: human peripheral blood mononuclear cells; LRRFIP-1: leucine-rich repeat flightless-interacting protein-1; HaCat: human keratinocyte cell line; IG: intra-gastric; ASC: apoptosis-associated Speck-like protein Casp1: caspase-1; SGBS: Simpson–Golabi–Behmel syndrome; ALP: alkaline phosphatase; CCL-3: chemokine (C-C motif) ligand-3; TC: total cholesterol, TAG: triacylglycerol; LDL-C: low-density lipoprotein cholesterol; TAC: total antioxidant capacity; MDA: malondialdehyde; SOD: superoxide dismutase; CAT: catalase; GPX: glutathione peroxidase; GSH: glutathion, OA: osteoarthritis; COX2: cyclooxygenase-2; PGE2: prostaglandin-E2; EGCG: epigallocatechin-3-*O*-gallate; MMP-2: matrix-degrading enzyme metalloproteinase; NADPH oxidase: nicotinamide adenine dinucleotide phosphate oxidase; CXCL-10: C-X-C motif ligand 10; M-CSF: macrophage colony-stimulating factor; PPARγ: peroxisome proliferator-activated receptor; NFE2L2: nuclear factor, erythroid 2 like 2; TRXR2: thioredoxin reductase 2; MnSOD: manganese superoxide dismutase; TXNIP: thioredoxin-interacting protein; NLRP3: NOD-like receptor (NLR) family, pyrin domain containing 3; PPARα: peroxisome proliferator activated receptor-α; CPT-1: carnitine palmitoyl transferase-1; SREBP-1: sterol regulatory element binging protein 1; SCD-1: stearoyl-CoA desaturase-1; vEGF: vascular endothelial growth factor; eNOS: endothelial nitric oxide synthase; PGC-1α: peroxisome proliferator-activated receptor coactivator 1α; GLUT-4: glucose transporter-4; VCAM-1: endothelial vascular cell adhesion molecule-1; PXR: pregnane X receptor; GST: glutathione S-transferase; MDRP1: multidrug-resistant protein 1, CYP1A1: cytochrome P450; SIRT1: sirtuin 1; ↑: increase; ↓: decrease.

**Table 5:** Polyphenol metabolites and functions.

Polyphenols	Subclasses	Metabolites	Bacterial Catabolism	Metabolites Functions	Ref
Flavonols 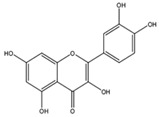	Quercetin	3,4-DHPAA 3-HPAA 4-HPAA	*Clostridium orbiscidens Eubacterium oxidoreducens* *Eubacterium ramulus* *Enterococcus casseliflavus*	Oxygen radical scavenging (all the metabolites), SOD- like activities (3,4 DHPAA), ↑glutathione S-transferase (3,4 DHPAA), ↑Nrf2-AhR (3,4 DHPAA) ↓Proinflammatory cytokines (3,4 DHPAA) ↑Glucose induced-insulin secretion (3,4 DHPAA) ↑Function and survival of pancreatic β-cells (3,4 DHPAA) Protective effect against OxS induced-endothelial dysfunction (3,4 DHPAA)	[132,208,209,210,211,212,213,214,215,216,217,218,219,220]
Flavones 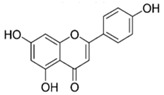	Apigenin	Phloretin 3-HPPA 4-HPPA 4-HCA	*Clostridium orbiscindens*	↓Oxygen radical scavenging (3-HPPA) ↓Proinflammatory cytokines (3-HPPA) ↑Glucose induced-insulin secretion (3-HPPA) ↑Function and survival of pancreatic β-cells (3-HPPA) Protective effect against OxS induced-endothelial dysfunction (3-HPPA)	[132,208,209,212,213,214,215,221]
Flavanones 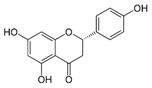	Naringenin	3,4-DHPPA 3-HPPA 4-HPPA	*Clostridium* strains *Eubacterium ramulus*	↓Oxygen radical scavenging (3-HPPA) ↓Proinflammatory; 3,4 DHPPA) ↑Glucose induced-insulin secretion (3-HPPA) Protective effect against OxS induced-endothelial dysfunction (3-HPPA)	[34,60,209,212,213,214,215,217,222,223]
Isoflavones 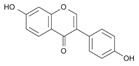	Daidzein	(S)-Equol *O*-DMA	*Bacteroides ovatus*, *Streptococcus intermedius*, *Ruminococcus productus*, *Eggerthella* sp.Julong 732, *Enterococcus faecium* EPI1, *Lactobacillus mucosae* EPI2, *Finegoldia magna* EPI3 *Clostridium* spp. HGHA136	Stimulation of cellular antioxidant systems ↑Catalase and SOD activity Anti-atherogenic effect	[224,225,226]
Flavan-3-ols 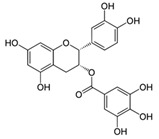	Monomers (catechins, epicatechins) and proanthocyanidins	3-HPPA 3,4-DHPPA 3′,4′-DHPVL 3,4-DHPVA 3′-HPVL 3′,4′,5′-THPVL 3′,5′-DHPVL	*Clostridium coccoides*, *Bifidobacterium* spp. *Eggerthella lenta* *Flavonifractor plautii*	↓Oxygen radical scavenging (3-HPPA) ↓ROS generation (3′-HPVL, 3′,4′-DHPVL) ↓NF-κB transcriptional activity ↓NO synthesis (3′,4′,5′-THPVL; 3′,4′-DHPVL) ↓iNOS expression (3′,4′-DHPVL) Maintenance of endothelial homeostasis and functions (3′,4′-DHPVL): ↓Endothelial adhesion (3′,4′-DHPVL) ↓VCAM1 and MCP1 (3′,4′-DHPVL) ↓Systolic blood pressure (3′,4′,5′-THPVL; 3′,5′-DHPVL)	[87,92,109,131,227,228,229,230,231,232,233,234,235,236]
Anthocyanins 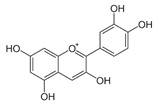	Cyanidin Peonidin Pelargonidin Malvidin Delphinidin	Protocatechuic acid Vanillic acid 4-Hydroxybenzoic acid Syringic acid Gallic acid	*Lactobacillus plantarum*, *Lactobacillus casei* *Lactobacillus acidophilus* LA-5 *Bifidobacterium lactis* BB-12	Antidiabetic activities due to their antioxidant capacity ↓DNA damages, ↓ROS production ↑Cellular glutathione level, ↑glucose uptake by HepG2 and human skeletal cells, ↑glycogen production by HepG2 cells, ↑Mitochondria homeostasis	[237,238,239,240,241,242]
Hydroxycinnamic acids 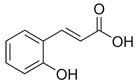	Chlorogenic acids	3-HPPA 3,4-DHPPA Caffeic acid	*Escherichia coli, Bifidobacterium lactis*, *Lactobacillus gasseri*	↓Oxygen radical scavenging(3-HPPA) ↓Proinflammatory cytokines (3-HPPA; 3,4 DHPPA) Antidiabetic activities due to its antioxidant capacity (caffeic acid): ↑Cellular glutathione level ↓DNA damages ↓Cytotoxicity, ↓ROS production ↑Glucose consumption ↑Glycogen production	[209,212,214,215,243,244]
Hydrolyzables tannins 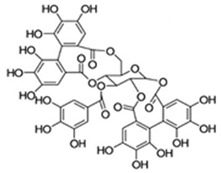	Ellagitannins	Ellagic acid Urolithin A Urolithin B	*Butyrivibrio* spp.	↓Intracellular ROS accumulation (Urolithin A) ↓Cellular injury by ROS ↓Proinflammatory mediators (Ellagic acid and Urolithin A) ↓NADPH oxidase activation (Urolithin A) ↓PGE2 production (Urolithin A and B) ↓mPGES-1 and COX-2 expression (Urolithin A and B) ↓Proteins glycation (Urolithin A and B) ↓Triglycerides accumulation (Ellagic acid and Urolithin A) ↓Expression of adipogenic protein and gene (Urolithin A) ↑Fatty acid β-oxidation (Urolithin A) Alleviation of myocardial ischemia/reperfusion injury (Urolithin A)	[245,246,247,248,249,250,251,252,253,254,255,256,257]
Lignans 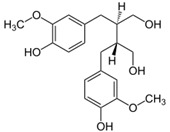	Secoisolariciresinol	Enterodiol Enterolactone	*Bacteroides distasonis*, *Bacteroides fragilis*, *Bacteroides ovatus*, *Clostridium cocleatum*, *Butyribacterium methylotrophicum*, *Eubacterium callanderi*, *Eubacterium limosum*, *Peptostreptococcus productus*, *Clostridium scindens*, *Eggerthella lenta*	Antioxidant capacity OH-scavenging activity Immunomodulatory effects in human cells ↓NF-κB transcriptional activity ↓Proinflammatory cytokines expression	[258,259,260,261]
Stilbenes 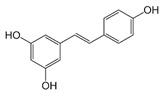	Trans-resveratrol	DHR 3,4′-dihydroxy-trans-stilbene 3,4′-dihydroxybibenzyl (lunularin)	*Slackia equolifaciens* *Adlercreutzia equolifaciens*	Antioxidant activity Free radical scavenging (DHR) ↓NO production (DHR)	[262,263,264]

3,4-DHPPA, 3,4-dihydroxyphenylpropionic acid; 3-HPPA, 3-hydroxyphenylpropionic acid; 4-HPPA, 4-hydroxyphenylpropionic acid; 4-HCA, 4-hydroxycinnamic acid; 3,4-DHPAA, 3,4-dihydroxyphenylacetic acid; 3-HPAA, hydroxyphenylacetic acid; 4-HPAA,4-hydroxyphenylacetic acid. *O*-DMA, O-demethylangolensin, 3,4-DHPVA, 3,4-dihydroxyphenyl-γ-valeric acid; 3′,4′,5′-THPVL, 3′,4′,5′-trihydroxyphenyl-γ valerolactone; 3′,4′-DHPVL, 3′,4′-dihydroxyphenyl-γ-valerolactone; 3′,5′-DHPVL, 3′,5′-Dihydroxyphenyl-γ-valerolactone 3′,-HPVL, 3′-hydroxyphenyl-γ-valerolactone; DHR, dihydroresveratrol, NO: nitric oxide; ↑: increase; ↓: decrease.

## Abbreviations

CMD: cardiometabolic disorders; CVD: cardiovascular disease; DAPP: dried apple peels; ERK: extracellular regulated kinases; GI: gastrointestinal; HPAA: phenylacetic acids; HPPA: phenylpropionic acid; IL: interleukin; IPEC: intestinal porcine enterocyte cell line; IR: insulin resistance; MetS: Metabolic syndrome; miRNAs: micro-RNAs; NF- κB: nuclear factor-kappa B; Nrf2: nuclear redox factor-2; OxS: oxidative stress; ROS: reactive oxygen species; TNFα: tumor necrosis factor-alpha.
